# Correction: Exciton and charge transfer processes within singlet fission micelles

**DOI:** 10.1039/d5sc90113h

**Published:** 2025-05-28

**Authors:** Daniel Malinowski, Guiying He, Bernardo Salcido-Santacruz, Kanad Majumder, Junho Kwon, Matthew Y. Sfeir, Luis M. Campos

**Affiliations:** a Department of Chemistry, Columbia University New York New York 10027 USA lcampos@columbia.edu; b Department of Physics, Graduate Center, City University of New York New York NY 10016 USA msfeir@gc.cuny.edu; c Department of Chemistry, Graduate Center, City University of New York New York NY 10016 USA; d Photonics Initiative, Advanced Science Research Center, City University of New York New York NY 10031 USA

## Abstract

Correction for ‘Exciton and charge transfer processes within singlet fission micelles’ by Daniel Malinowski *et al.*, *Chem. Sci.*, 2025, https://doi.org/10.1039/d5sc01479d.

The authors regret that the funding information provided in their published article is incorrect. The correct National Science Foundation grant numbers are DMR-2453907 and DMR-2453908.

The Royal Society of Chemistry apologises for these errors and any consequent inconvenience to authors and readers.

